# Wild bee diversity in the village of Peyresq and the surrounding areas in the southern French Alps

**DOI:** 10.3897/BDJ.14.e213911

**Published:** 2026-10-27

**Authors:** Jordan Benrezkallah, Thomas Brau, Frédéric Carion, Bastien De Tandt, Rémi Devorsine, Luca Dorio, Thierry Duez, Dimitri Evrard, Simone Flaminio, Guillaume Ghisbain, Arthur Herinckx, Clara Leconte, Shawn Lemaire, Anatole Maugendre, Denis Michez, Alice Naveaux, Sara Reverte, Blandine Rigaut, Rémi Santerre, Clément Tourbez, Jean Van den Abeele, Pol Vannechel, Maxence Gérard

**Affiliations:** 1 Laboratory of Interaction Ecology and Global Change, Research Institute for Biosciences, University of Mons, Place du parc 20, Mons, Belgium Laboratory of Interaction Ecology and Global Change, Research Institute for Biosciences, University of Mons, Place du parc 20 Mons Belgium; 2 Laboratory of Zoology, Research Institute for Biosciences, University of Mons, Place du parc 20, Mons, Belgium Laboratory of Zoology, Research Institute for Biosciences, University of Mons, Place du parc 20 Mons Belgium; 3 Spatial Epidemiology Lab (SpELL), Université Libre de Bruxelles, 1000, Brussels, Belgium Spatial Epidemiology Lab (SpELL), Université Libre de Bruxelles, 1000 Brussels Belgium

**Keywords:** Conservation, France, Mountain Ecosystem, Occurrence, Wild Bees

## Abstract

Background

Wild bees are essential pollinators, yet the populations of an increasing number of species are declining across Europe, with knowledge gaps particularly acute in mountain regions. The southern French Alps represent a biogeographically exceptional area, situated at the interface of Alpine and Mediterranean influences, but their bee fauna remains poorly documented. Here, we present a curated dataset of wild bee occurrence records from the village of Peyresq (France) and its surroundings, collected over four consecutive years during summer along an elevational gradient spanning montane, subalpine and alpine zones.

New information

The dataset comprises 4,502 wild bee specimens, of which 4,007 specimens were identified to species level, representing 241 species across six families. The dataset includes six threatened species according to the European Red List of Bees, five being Vulnerable and one Endangered, as well as several species characteristic of Alpine and Mediterranean mountain faunas. A sampling completeness analysis estimated observed richness at about 88% of the predicted local bee fauna detectable during our sampling period (early to mid-July). All specimen data have been digitised following the Darwin Core standard, deposited in the collection of the University of Mons (Belgium) and made openly accessible through GBIF. This dataset constitutes a valuable baseline for biogeographical research and conservation planning in mountain ecosystems facing ongoing environmental change.

## Introduction

Animals pollinate over 80% of flowering plants, with wild bees recognised as amongst the most efficient pollinators supporting agricultural productivity and ecosystem stability (Ollerton et al. 2011). Despite their ecological importance, the populations of a large number of bee species are declining globally, primarily driven by anthropogenic factors including habitat loss, agricultural intensification, pesticide use, the expansion of invasive species and climate change (Brown and Paxton 2009, Potts et al. 2010, Potts et al. 2016, Kammerer et al. 2021, Gekière et al. 2025). In this context, species checklists and IUCN Red Lists are essential tools for monitoring biodiversity trends and prioritising conservation efforts to counteract these declines. In Europe, nearly 2,200 bee species have been recorded (Ghisbain et al. 2025), of which > 500 are declining and > 1,100 have an unknown population trend according to the most recently published European Red List of bees (Michez et al. 2026). Moreover, the high proportion of Data Deficient species (14.3%) — i.e. species that cannot be assessed due to a lack of information on their distribution, taxonomy and/or population status — underscores significant knowledge gaps, particularly in mountain regions where distributional data remain scarce. This issue is further compounded given that > 20% are endemic to Europe, many of which are confined to Mediterranean islands and montane habitats (Michez et al. 2026).

Following the publication and data of the latest Red List, the “Assessment to Planning” (A2P) report of the IUCN identified mountain biomes as particularly vulnerable for bee populations (Reverté et al. 2026). These environments host distinct montane, subalpine and alpine communities, characterised by unique floral and faunal assemblages. In the high mountains of southern Europe, most bee groups decrease in diversity with elevation, with the notable exception of bumblebees (*Bombus* spp.), which dominate alpine communities (Hoiss et al. 2012, Minachilis et al. 2020). Importantly, mountain bee communities can exhibit a high degree of ecological specialisation along elevational gradients (Osorio-Canadas et al. 2021). While such specialisation allows them to exploit specific ecological niches, it may also increase their vulnerability to environmental change, as shifts in climate and habitat conditions can disproportionately affect these species (Hoiss et al. 2012). Consequently, mountain ecosystems, already highly susceptible to climate change and other anthropogenic pressures, may represent hotspots of risk for high-altitude bees.

Mainland France hosts > 980 bee species (Ropars et al. 2025) and encompasses several major mountain ranges, including the Alps, Pyrenees and Massif Central, which harbour particularly vulnerable bee communities (Michez et al. 2026). The high proportion of Data Deficient species amongst the French fauna underscores substantial knowledge gaps that limit our ability to accurately assess species distributions and implement effective conservation strategies (Michez et al. 2026). Recent inventories in several Alpine subregions reveal substantial and still-growing bee diversity. In Switzerland, > 630 species have been recorded, with diversity hotspots located in xeric inner-Alpine valleys hosting unique sub-Mediterranean and subalpine assemblages (Praz et al. 2023). Ongoing taxonomic work continues to uncover previously overlooked diversity, as illustrated by the recent description of *Andrena amieti*, a widespread Alpine species (Praz et al. 2019). The southern French Alps are particularly promising in this regard, given their exceptional biogeographical position at the crossroads of Alpine and Mediterranean influences, their remarkable floristic richness and their high levels of plant endemism (Pawłowski 1970, Casazza et al. 2005, Médail and Diadema 2006, Carles and Thébault 2017), conditions expected to support high bee diversity. Yet, this region remains strikingly under-inventoried: aside from the historical work of Benoist (1928) and Schmid-Egger (2011), it has received only limited attention, with few recent taxonomic studies (Meunier et al. 2023, Aubert et al. 2024, Müller et al. 2024). To help address these knowledge gaps, this paper presents bee occurrence data from the village of Peyresq and its surrounding areas in the southern French Alps (Fig. 1), a region whose location at the interface of Alpine and Mediterranean influences renders it particularly valuable for ecological and conservation research.

## Material and methods

### Geographic coverage

#### Description

The bees were collected in the south-eastern French Alps, within the Departments of Alpes-Maritimes and Alpes-de-Haute-Provence. A total of five municipalities were sampled across an elevational gradient from 910 to 2,474 m, with a strong focus on the village of Peyresq (Municipality of Thorame-Haute) (see Fig. 1 for site locations). Sampling sites were selected, based on their accessibility from the village of Peyresq, including hiking trails, roadside habitats and other areas identified via on-site observations and local recommendations. Several of our sites are near the boundary of the Mercantour National Park, a protected area whose bee fauna was inventoried by Schmid-Egger (2011) (see Discussion). Sampling along the altitudinal gradient covers three main elevation levels (Ozenda 1963): the montane level (ca. 910–1,600 m in our study), characterised by mixed broadleaf and coniferous forests (notably *Fagus sylvatica* and *Pinus sylvestris*); the subalpine zone (ca. 1,600–2,200 m in our study), dominated by coniferous forests, primarily *Larix decidua*; and the alpine zone (above 2,200 m up to 2,474 m in our study), consisting of open habitats, such as alpine grasslands above the treeline. The altitude of each elevational level varies depending on several factors, such as its slope aspect (e.g. north- versus south-facing). Within each elevational level, sampling sites were distributed across slopes and solar exposure to account for local topoclimatic variability. Human pressure in the study area is low (e.g. no intensive agriculture, very small resident population), but the landscape has been shaped by rural exodus: fields around Peyresq were abandoned in the 1950s and montane meadows are progressively replaced by forest (Gassendi 1992, Navello-Sgaravizzi 1992). At some sites, this decline in grazing and the resulting closure of montain grasslands may be detrimental to bees, as open, early-successional habitats give way to shrubland and woodland (Penado et al. 2022). Subalpine and alpine grasslands, in contrast, are still grazed by transhumant sheep herds during summer.

#### Coordinates

Latitudes: 43.97036-44.30028

Longitudes: 6.506148-6.711395

### Temporal coverage

Field missions were conducted annually over four consecutive years (2022–2025), each lasting approximately 10 days in early July.

Sampling dates ranged as follows:

- 2022-07-05 – 2022-07-12
- 2023-07-05 – 2023-07-12
- 2024-07-09 – 2024-07-17
- 2025-07-09 – 2025-07-14

**Notes**: Sampling effort varied over time according to the needs of ongoing projects. The number of bees captured per sampling day ranged between 2 and 780, with an average of 150 and a median of 105.

### Sampling methods

Each year, sampling was conducted by a team of 7–9 people affiliated with the Laboratory of Zoology at the University of Mons (Belgium), including five Master’s students and their supervisors. Participants had varying levels of experience in entomological fieldwork, ranging from novices to experts. Bees were collected opportunistically using nets, targeting a wide variety of flowering plants to maximise species diversity and abundance. After collection, specimens were euthanised, pinned and labelled with standard collection information, including date, geographic coordinates, locality name and altitude. Specimens were stored at ambient temperature before identification.

### Specimen identification

Species names follow the taxonomic backbone by Ghisbain et al. (2025), with a few exceptions where more recent revisions were applied. Species-level identification was performed by Achik Dorchin (Eucerini), Anatole Maugendre (Anthidiini), Clément Tourbez (Megachilidae), Rémi Devorsine (Xylocopinae), Frédéric Carion (*Nomada*), Simone Flaminio (Halictidae), Thomas Brau (Halictidae), Guillaume Ghisbain (*Bombus*), Romain Le Divelec (*Hylaeus*), Petr Bogusch (*Sphecodes*), Jakub Straka (*Sphecodes* and *Nomada*), Thomas James Wood (Andrenidae), Rémi Santerre (*Anthophora*, *Ceratina*, *Eucera*, *Xylocopa*), Maxence Gérard (*Melitta*, *Panurgus*, *Tetralonia*, *Xylocopa*), Denis Michez (*Melitta*) and Blandine Rigaut (*Dufourea*, *Hylaeus* and *Sphecodes*). Full details on identifiers and their respective taxonomic assignments are provided in the associated dataset. Following identification, a label indicating the species name, identifier and sex was pinned. All specimen data were digitised using the Darwin Core data standard (Wieczorek et al. 2012) and specimens were deposited in the collection of the University of Mons (Belgium). Two taxa could not be identified to species level. Bombus sensu stricto subgenus cannot be reliably separated on morphological criteria; they were therefore recorded as *Bombus* s. str. spp. Specimens of Halictus aff. adjikenticus are morphologically close to this taxon, but differ from known populations of *H. adjikenticus*. As the subgenus Halictus (Monilapis) is a complex group in which females are often indistinguishable, a revision is necessary to correctly identify these specimens (Brau et al., in prep). These two taxa were retained in the dataset, but are not counted as species in the totals below.

### Map processing

The map of sampling sites in the southern Alps was produced using QGIS v.3.36. Administrative boundaries were obtained from Urbano (2018)), elevation data from OpenTopography (2021) and hydrographic data from DREAL Provence-Alpes-Côte d'Azur (2017). Projection map:RGF93 / Lambert Conformal Conic (EPSG:2154). Species distribution maps were generated from occurrence records compiled by Sentil et al. (2026) using R v.4.3.1 with the packages giscoR (Hernangómez 2026), sf (Pebesma and Bivand 2023) and ggplot2 (Wickham 2016). Administrative boundaries for European distribution maps were obtained from Eurostat GISCO (contains administrative boundaries © EuroGeographics). These occurrence records cover only Europe; consequently, maps for species whose range extends beyond Europe (e.g. to North Africa, Asia Minor or the Levant) are incomplete and should be interpreted as representing only the European portion of the species' range.

## Data resources

The complete dataset is publicly accessible in an updatable version available online at https://doi.org/10.15468/9zvkch (Benrezkallah 2026). Note that the data collected in 2022 were previously published in Sentil et al. (2026).

## Results and Discussion

### Taxonomic coverage

#### Diversity and abundance of species

During the four years of the survey, 4,502 bee specimens were collected across 243 taxa, representing six families: Andrenidae (39 species, 565 specimens), Apidae (52 species, 2,194 specimens), Colletidae (17 species, 152 specimens), Halictidae (62 species, 787 specimens), Megachilidae (71 species, 794 specimens) and Melittidae (1 species, 10 specimens). Of these, 496 specimens (11%) could not be identified to species level: 417 *Bombus* s. str. spp. and 79 Halictus aff. adjikenticus (see Specimen identification). The remaining 4,007 specimens were identified to 241 species. At the genus level, *Bombus* was the most abundant genus (1,954 specimens, of which 417 *Bombus* s. str. spp.), followed by *Lasioglossum* and *Andrena* (460 and 427 specimens, respectively; Fig. 2). The high number of *Bombus* specimens partly reflects a targeted sampling effort in 2025. More broadly, sampling effort and focus varied across years depending on the objectives of ongoing projects, so abundance data should be interpreted with caution when comparing across genera or years. The full species-level breakdown of abundance is provided in the Pareto chart (Fig. 2) and in the species checklist (Table 1).

To assess sampling completeness, species accumulation curves were constructed, and the Chao1 estimator was calculated following the method proposed by Chao et al. (2014). The Chao1 estimator is an abundance-based estimator that accounts for unseen species using the frequency of rare taxa. Based on the full dataset (2022–2025), the Chao1 estimator predicted a total species richness of 271.9 species, with a 95% confidence interval ranging from 248.2 to 295.7 species (Fig. 3), suggesting that the observed richness of 241 species represents approximately 88.6% of the estimated bee fauna detectable during our sampling period (mid-July, approximately ten days per year). This figure should nevertheless be interpreted with caution. As sampling was restricted to a single period of the season, the accumulation curve and the resulting estimate only reflect species flying in mid-July. Species with a spring or autumn phenology, which had no chance of being detected, are not accounted for by the estimator, so the total richness of the study area is likely higher than 275 species. Year-round sampling would be needed to capture the full bee fauna.

Our inventory and the one reported for the Mercantour National Park by Schmid-Egger (2011) overlap widely. The differences probably reflect mainly the sampling design: the Mercantour study used pan traps, covered a wider altitudinal range (400-2,600 m, compared to mainly 1,300-2,100 m in our study) and extended over a longer period, including June, whereas our sampling was limited in mid-July and did not use pan traps. This may explain why several small-bodied species (e.g. *Hylaeus*, *Ceratina*) and species restricted to high or low elevations in the region (e.g. *Colletes impunctatus*, *Hylaeus nivalis*, *Coelioxys argentea*) were recorded only in the Mercantour. On the other hand, our dataset includes 25 *Bombus* species, compared to 12 in the Mercantour inventory. This difference probably reflects our particular focus on bumblebees during sampling and the large number of grassland sites sampled, rather than necessarily a richer bumblebee fauna. Our dataset also includes several species that were abundant in our samples, but absent from the Mercantour inventory (e.g. *Chelostoma foveolatum*, *Andrena labialis*, *Bombus barbutellus*).

### Threatened and protected species

#### Species threatened

Of the 241 species identified, six are classified as threatened according to the European Red List of Bees (Michez et al. 2026), comprising one Endangered (EN) and five Vulnerable (VU) species, accounting for 2.8% of the total species collected. An additional nine species were classified as Near Threatened (NT) and three as Data Deficient (DD) (Table 1).

Mountain bees are particularly vulnerable to climate change, as cold-adapted species are forced to shift upslope, with limited possibilities of dispersal between isolated massifs. They are also affected by land-use change, through both overgrazing and habitat closure following pastoral abandonment (Reverté et al. 2026). The occurrence of threatened species in the study area underlines the importance of maintaining open, flower-rich grasslands through moderate grazing.

For each of the six threatened species, a brief overview of their diagnosis, ecology and conservation status is provided below, along with photographic documentation and a distribution map at the European level, to contextualise the significance of their occurrence in the study area (Figs 4 – 9). Distribution maps are based on occurrence records available in Sentil et al. (2026), white dots indicate records with no collection year, dark blue dots indicate records collected before 1990 and light blue dots indicate records collected from 1990 onwards, a threshold chosen to reflect the increase in systematic naturalist surveys across Europe from that period.

***Bombus pomorum* (Panzer, 1805) - VU**

**Diagnosis**: *Bombus pomorum* is superficially similar to several sympatric species that share a predominantly black colour pattern with a red tail. It can be distinguished from Bombus (Melanobombus) lapidarius, B. (Alpigenobombus) mastrucatus and B. (Kallobombus) soroeensis proteus by examining the posterodistal edge of the mesobasitarsus: this edge is acute (spine-like) in *B. pomorum*, but blunt (rounded) in the other species. When this character is difficult to observe, *B. pomorum* can also be recognised by its distinctly swollen metatibia, whereas it is comparatively flatter in other species. It can be separated from B. (Thoracobombus) ruderarius (which also exhibits an acute posterodistal edge of the mesobasitarsus and a swollen metatibia) by the shape of the labral lamella, which is acute in *B. pomorum* and bead-shaped in *B. ruderarius*. In addition, *B. pomorum* has a relatively longer oculo-malar distance and a more distinctly tabular clypeus (i.e. bulging basally and flattened apically).

**Notes**: This species (Fig. 4) was historically widespread across Europe, ranging from western France to eastern part of Russia (up to the Ural Mountains) and from southern Sweden to Greece (Reverté et al. 2023, Sentil et al. 2026). However, it has been extirpated from a large portion of its former range (Rasmont et al. 2021). Females are primarily associated with Fabaceae, while males are frequently observed visiting Cardueae (Folschweiller 2020). *Bombus pomorum* is currently classified as Vulnerable on the European IUCN Red List of Bees (Michez et al. 2026).

***Nomada rhenana* Morawitz, 1872 - VU**

**Diagnosis**: *Nomada rhenana* belongs to the subgenus *Nomada* Scopoli, 1770 (Straka et al. 2024). Based on Smit (2018), the females of *Nomada rhenana* can be identified in the region by this combination of characters: Apex of the mandibles simple and rather sharp; longitudinal carina between the antennae sharp; malar area short; labrum entirely punctate and with one labral tooth just below the centre; third antennal article as long as the fourth; procoxa simple; metatibia apically with four rather short spines; hairs of the scutum short and decumbent; scutellum shiny with interspaces going up to three punctures width; propodeum distinctly and closely punctate on the sides; fifth sternum without longitudinal carina. Males can be identified by this combination of characters: Apex of the mandibles simple and sharp, without additional tooth; longitudinal carina between the antennae sharp; malar area shorter than the width of an antenna; head not greatly protruding; labrum entirely punctate with one strong tooth just below the centre; third antennal article about three quarters the length of the fourth; antennal articles without any carina nor tubercles; metafemur basally flattened for one third and covered with short hairs, except basally where some longer hairs are present, hairs never forming a golden hair tuft; metatibia apically with three to four short spines; seventh tergum rounded or indented.

**Notes**: This species (Fig. 5) is widely distributed across Europe, ranging from the southern Iberian Peninsula up to Belarus and the region of Bashkortostan in Russia (Reverté et al. 2023, Sentil et al. 2026). *Nomada rhenana* parasitises the mining bees *Andrena gelriae* van der Vecht, 1927 and *Andrena ovatula* (Kirby, 1802) (Smit 2018, Straka et al. 2024). *Nomada rhenana* is currently classified as Vulnerable on the European IUCN Red List of bees (Michez et al. 2026).

***Halictus carinthiacus* Blüthgen, 1936 - EN**

**Diagnosis**: *Halictus carinthiacus* belongs to the subgenus *Monilapis* Cockerell, 1931 (Pesenko 2004). *Halictus carinthiacus* is a highly characteristic species of the subgenus *Monilapis*. The female, characteristic of alpine regions, can be distinguished from other sympatric *Halictus* species by the strongly developed vertex, the tergal hair bands that cover only half of the apical margin medially and the slightly matt interspaces between the punctures of the tergites. In addition, it is generally larger than other species of Halictus (Monilapis). The male shares the diagnostic characters of the female and can additionally be recognised as a member of *Monilapis* by the genal area, which is laterally concave and carinate, as well as by the upper gonostylus bearing a pencil of long hairs arising from the middle of its dorsal surface. It differs from *H. simplex* Blüthgen, 1923, with which it shares certain similarities, by its larger size, the very short moniliform antennae and the upward-directed tuft of hairs on the upper gonostylus.

**Notes**: The species (Fig. 6) is distributed throughout the Alps and adjacent regions, ranging from France to Croatia (Reverté et al. 2023, Sentil et al. 2026). It occurs primarily in montane and subalpine zones, inhabiting open to semi-open, thermophilic environments (Brau, pers. comm.). *Halictus carinthiacus* is currently classified as Endangered on the European IUCN Red List of Bees (Michez et al. 2026).

***Lasioglossum euboeense* (Strand, 1909) - VU**

*Diagnosis: Lasioglossum euboeense* belongs to the subgenus *Sphecodogastra* Ashmead, 1899. The female can be recognised by its rounded head and the labrum, which bears a distinct tubercle. The scutum is densely and finely punctate. The mesopleurae are alveolate anteriorly and longitudinally ridged posteriorly. The terga are densely and finely punctate. The male has an elongated head with a yellow labrum. The antennal articles are elongated, approximately 1.25 times longer than wide. The scutum is densely and finely punctate, while the mesopleurae are finely wrinkled. The first three terga are reddish and densely and finely punctate.

**Notes**: This species (Fig. 7) is widely distributed across Europe, but its range is fragmented and highly localised (Reverté et al. 2023, Sentil et al. 2026). Outside Europe, it also occurs in North Africa (Algeria) and across south-western Asia, from the Levant (Lebanon) through Asia Minor to the Caucasus (Azerbaijan) and Iran (Strand 1909, Blüthgen 1924, Blüthgen 1931, Ebmer 1974a, Warncke 1975, Ebmer 1978, Ebmer 1988, Ebmer 1995). It is a thermophilic species typically associated with warm, dry, steppic environments, particularly dry grasslands and ruderal habitats (Brau, pers. comm.). *Lasioglossum euboeense* is currently classified as Vulnerable on the European IUCN Red List of Bees (Michez et al. 2026).

***Lasioglossum laeve* (Kirby, 1802) - VU**

**Diagnosis**: *Lasioglossum laeve* belongs to the subgenus *Sphecodogastra* Ashmead, 1899. The female can be recognised by its rounded head and the strongly punctate supraclypeal area. The scutum is densely punctate along the margins, but sparsely punctate medially, with smooth interspaces. The mesopleurae are distinctly reticulate and wrinkled. Terga 1–3 are smooth, with very sparse and fine punctation.The male likewise exhibits a rounded head and a dark labrum. It shares the same punctation pattern of the scutum and terga I–III as the female.

**Notes**: This species (Fig. 8) is widely distributed across Europe, but its range is fragmented and highly localised (Reverté et al. 2023, Sentil et al. 2026). Outside Europe, it also occurs in western and central Asia, from the Levant (Palestine, Israel, Syria) through Asia Minor to Armenia and Iran and as far east as the Altai Republic of Russia (Ebmer 1974b, Warncke 1975, Ebmer 1978, Warncke 1982, Ebmer 1995). *Lasioglossum laeve* appears to favour dry, mid-altitude habitats, with most records originating from dry meadows and ruderal areas. Pesenko (1999) regarded this species as primarily forest-dwelling; however, this does not seem to apply to populations in Switzerland (Amiet et al. 2001, Westrich 2019). *Lasioglossum laeve* is currently classified as Vulnerable on the European IUCN Red List of Bees (Michez et al. 2026).

***Trachusa interrupta* (Fabricius, 1781) - VU**

**Diagnosis**: *Trachusa interrupta* belongs to the subgenus *Paraanthidium*. While several species of *Trachusa* co-occur in the studied region, members of the subgenus *Paraanthidium* can be readily distinguished by the combination of numerous yellow maculations and the second recurrent vein in a postfurcal position, i.e. meeting the top of the second discoidal cell rather than the base of the second cubital cell. This species further differs from the very similar *T. integra* by the following characters: females possess dark brown to black pronotal lobes, usually with a low carina, whereas in males, the yellow tergal bands are medially interrupted on tergites I and II, while in *T. integra*, the interruption is restricted to tergite I (Kasparek 2022).

**Notes**: This species (Fig. 9) occurs mainly in the Mediterranean Region, from southern Portugal and North Africa to Greece and Türkiye, but also has populations in eastern European countries, such as Ukraine and Romania, as well as in central France (Kasparek 2020, Reverté et al. 2023, Sentil et al. 2026). It is specialised on Caprifoliaceae species (Muller 1996, Kasparek 2020). It is currently classified as Vulnerable on the European IUCN Red List of bees (Michez et al. 2026). The species is listed as a priority in European species action plan (Michez 2023).

#### Species typical of the region

Several species recorded in this study are characteristic of the southern Alpine and Mediterranean mountain fauna. Their European distributions are largely restricted to mountainous regions, occurring either in central and southern European mountain ranges (e.g. *Bombus mendax*, Fig. 10), discontinuously across southern mountain systems (e.g. *Nomada gransassoi*; Fig. 11), more broadly across Europe, but with a marked association with mountainous and Mediterranean areas (e.g. *Megachile parietina*; Fig. 12) or following a boreo-alpine distribution pattern (e.g. *Osmia inermis*; Fig. 13). Collectively, these species highlight the dual Alpine–Mediterranean character of the Peyresq area and emphasise its relevance as a study system for investigating bee distribution and ecology at the interface of two major European biogeographical regions.

***Bombus mendax* Gerstäcker, 1869**

**Diagnosis**: *Bombus mendax* is the only European representative of the subgenus *Mendacibombus* (Ghisbain et al. 2025). It can be readily distinguished from all other European species by the shape of its labrum, the tubercules of which are joined by a ridge that is complete (no median longitudinal furrow). The identification can be further confirmed by observing the outer surface of the metatibia, which is very coarsely sculptured and matt.

**Notes**: This strictly montane species (Fig. 10) is distributed across the Cantabrian Mountains, the Alps and the Pyrenees (Reverté et al. 2023, Sentil et al. 2026). In the Alps, it occurs at elevations ranging from 1,600 to 3,000 metres (Manino et al. 2007, Bossert 2014). The specimens collected in this study represent one of the southernmost known occurrences for alpine populations. *Bombus mendax* is associated with Fabaceae and is currently classified as Least Concern on the European IUCN Red List of Bees (Michez et al. 2026, Wood et al. 2021).

***Nomada gransassoi* Schwarz, 1986**

**Diagnosis**: *Nomada gransassoi* belongs to the subgenus *Nomada* Scopoli, 1770 (Straka et al. 2024). Based on Smit (2018), the females of *Nomada gransassoi* can be easily identified in the region by this combination of characters: Apex of mandibles bidentate; labrum basally impunctate; apex of metatibia with three to four brown spines clearly separated. On the other hand, males can be identified by this combination of characters: Apex of mandibles bidentate; apex of metatibia with three to four very short red spines; antennal articles squared, about as long as wide; labrum always yellow.

**Notes**: This species (Fig. 11) is restricted to montainuous habitat of northern Spain, Central Italy and the Alps (Reverté et al. 2023, Sentil et al. 2026). The host of *Nomada gransassoi* is currently unknown (Smit 2018, Straka et al. 2024). *Nomada gransassoi* is classified as Least Concern on the European IUCN Red List of bees (Michez et al. 2026).

***Megachile parietina* (Geoffroy, 1785)**

**Diagnosis**: *Megachile parietina* belongs to the subgenus *Chalicodoma* Lepeletier, 1841 (Praz 2017). The female can be readily distinguished from other *Megachile* species occurring in the region by its large size (16–18 mm), the entirely black body vestiture (except for the scopa, which may be orange) and the wings with a dark metallic sheen. Likewise, the male is characterised by its large size (14–17 mm) and by the tergal pilosity, with long brown hairs on tergites I–III and black hairs on tergites IV–VI, without distinct hair bands. Males further differ from the very similar *M. pyrenaica* and *M. rufescens* by the absence of a pre-apical groove or carina on the apical margin of sternite IV (Praz 2017).

**Notes**: *Megachile parietina* (Fig. 12) is mainly distributed in the Mediterranean Region, but also occurs in Germany, Austria, Czechia and around the Black Sea (Reverté et al. 2023, Sentil et al. 2026). The species is also present in Central Asia and the Middle East (Fateryga et al. 2018, Cassar 2024). It is specialised on Fabaceae with a strong affinity for *Onobrychis viciifolia* and usually builds its nest on rocky substrates (Cassar 2024). *M. parietina* is classified as Least Concern on the European IUCN Red List of bees (Michez et al. 2026).

***Osmia inermis* (Zetterstedt, 1838)**

**Diagnosis**: *Osmia inermis* belongs to the subgenus *Melanosmia* Schmiedeknecht, 1885 (Müller 2018). Females measure 8.5–9.5 mm in body length and can be distinguished from other small-sized *Melanosmia* species occurring in the region by the absence of hooked bristles on the galea and by the presence of a prominent tooth on the lower inner margin of the mandible. Similarly, males measuring 8.0–9.5 mm differ from related species by the absence of hooked bristles on the apical margin of sternite IV (Müller 2018).

**Notes**: This species (Fig. 13) is widely distributed across Europe, mainly in the northern part and mountainous regions, being distributed from northern Spain up to the Kirov Oblast in Russia and Fennoscandia (Reverté et al. 2023, Sentil et al. 2026). This species is polylectic, with visitation recorded on four plant families, but has a strong preference for Loteae (Fabaceae) (Müller 2018). *Osmia inermis* is classified as Least Concern on the European IUCN Red List of bees (Michez et al. 2026).

## Conclusions

This dataset provides a curated and standardised inventory of wild bees from the southern French Alps, a unique biogeographical region, yet insufficiently documented. Based on four consecutive years of sampling (2022–2025), it comprises 4,502 occurrences across 241 species and six families, representing a substantial contribution to the knowledge of bee diversity in mountainous areas at the interface between Alpine and Mediterranean Regions. The detection of six threatened species (one Endangered, five Vulnerable), nine Near Threatened and three Data Deficient species further emphasises the conservation relevance of this area and the importance of improving occurrence data in such environments.

We produced a high-quality dataset, formatted following the Darwin Core standard and openly available through GBIF, which constitutes a reliable baseline for future biogeographical and conservation studies. Yet, the dataset is characterised by heterogeneous sampling and non-standardised collection protocols across years, which naturally introduces biases in species detectability and relative abundance. In addition, the temporal coverage is restricted to a short period in early July, leading to an under-representation of species with different phenologies. Spatially, sampling was constrained to accessible areas around Peyresq, leaving parts of the surrounding region unexplored.

Future efforts should aim to extend both the spatial and temporal scope of sampling, particularly by including lower-elevation Mediterranean habitats and conducting surveys across a broader seasonal window. Expanding sampling into currently under-surveyed areas, including protected zones, such as the Mercantour National Park, would significantly improve regional coverage. Continued updates of this dataset will be essential to enhance its value for long-term biodiversity monitoring and to support conservation strategies in mountain ecosystems facing ongoing environmental change.

## Figures and Tables

**Figure 1.**
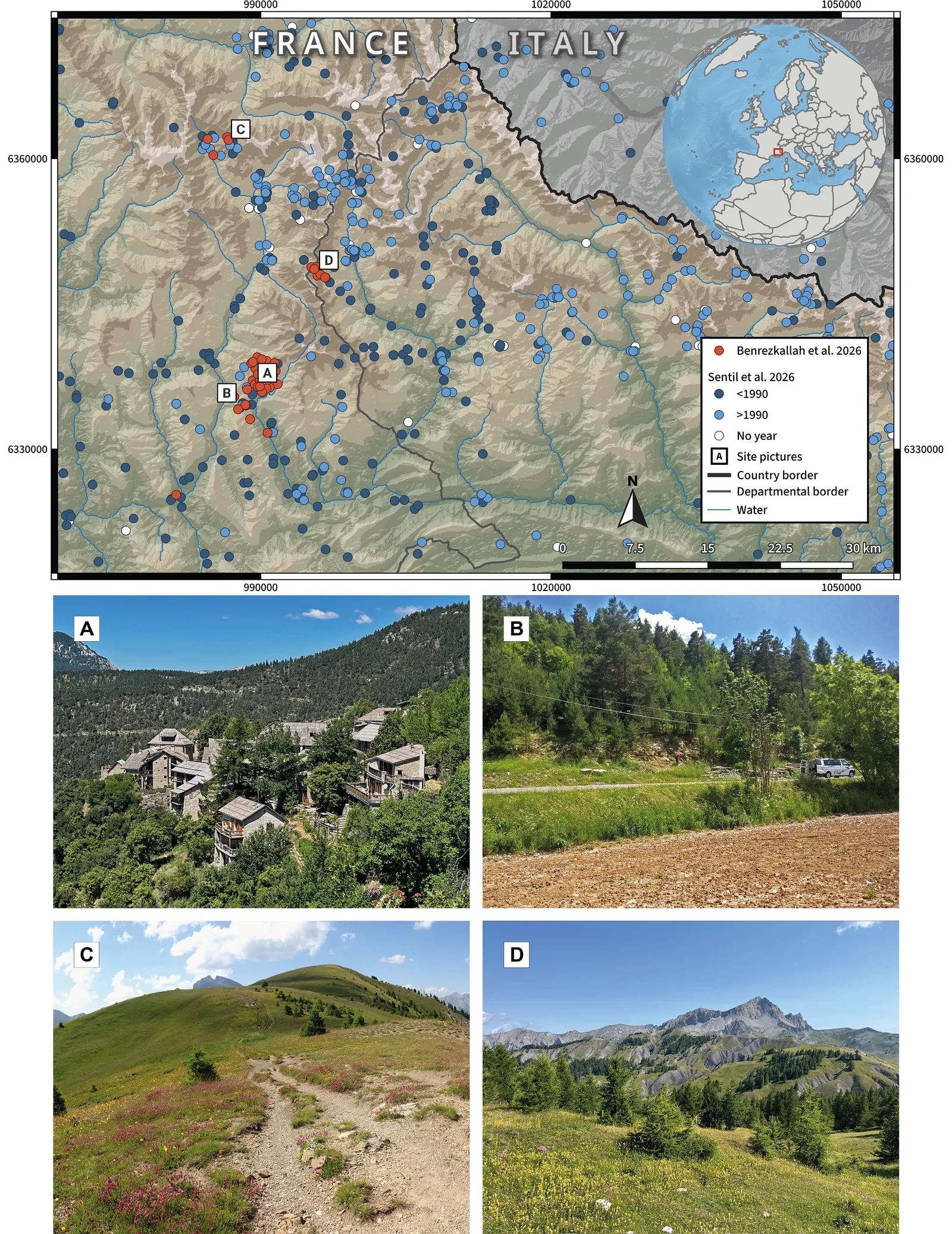
Map of sampling sites in the southern Alps. Data from this study are shown in red, while data from Sentil et al. (2026) are shown in blue. Lighter shades of blue indicate more recent sampling dates, with 1990 used as a threshold to reflect the marked increase in naturalist surveys across Europe during that decade; white dots indicate no year information. The number of records per period (Sentil et al. 2026 only) is: no year (n = 188), < 1990 (n = 761), ≥ 1990 (n = 3659). Sites labelled with letters on the map correspond to those illustrated in the pictures below. **A** Village of Peyresq (subalpine); **B** La Colle Saint-Michel (subalpine); **C** Col d’Allos (alpine); **D** Col des Champs (alpine). Photo credit: J. Benrezkallah (B, C) and G. Ghisbain (A, D). Map compiled by J. Benrezkallah.

**Figure 2.**
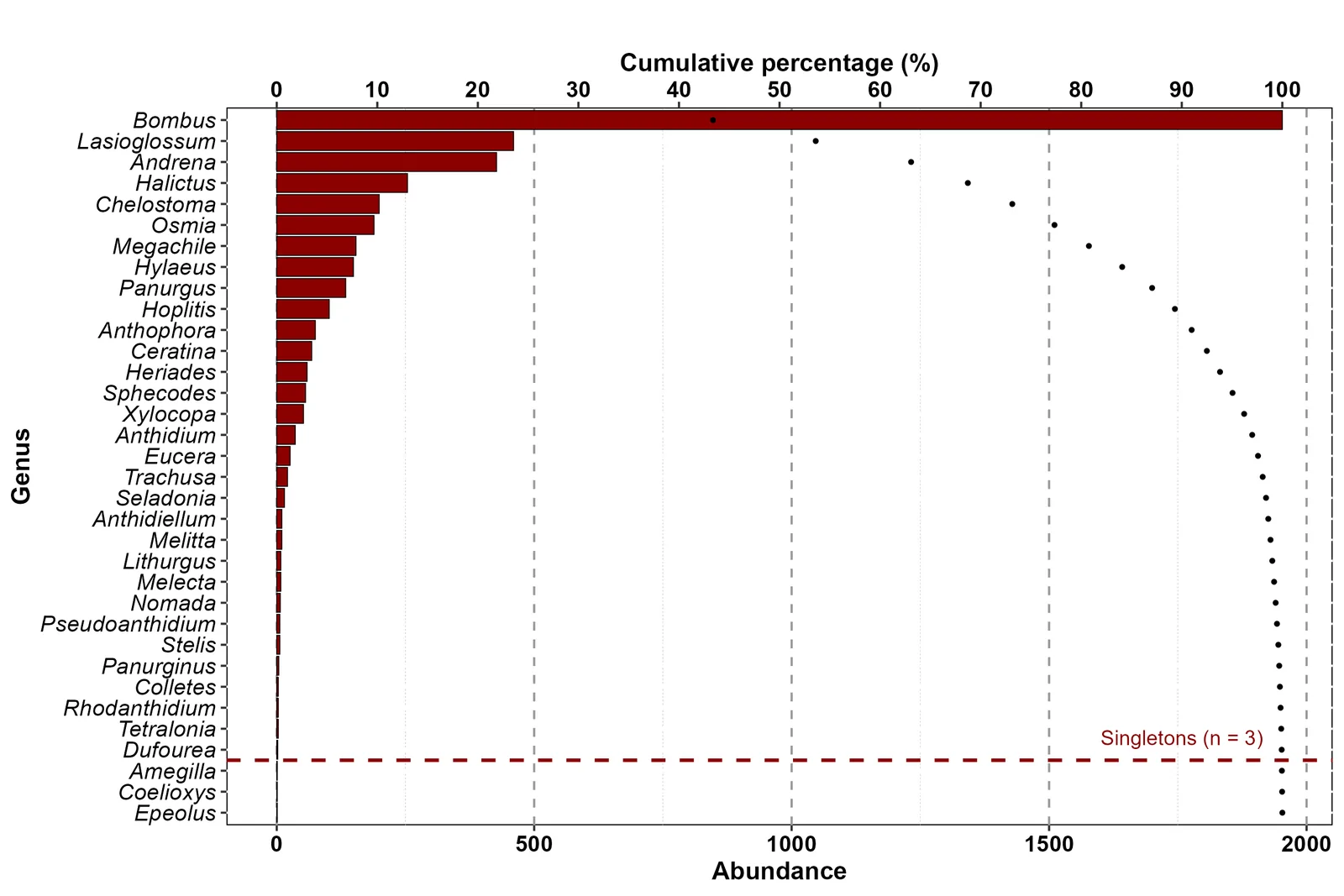
Pareto chart of bee abundance by genus. Bars represent the total number of individuals recorded per genus, ranked in descending order. Black dots indicate the cumulative percentage of the total number of collected individuals.

**Figure 3.**
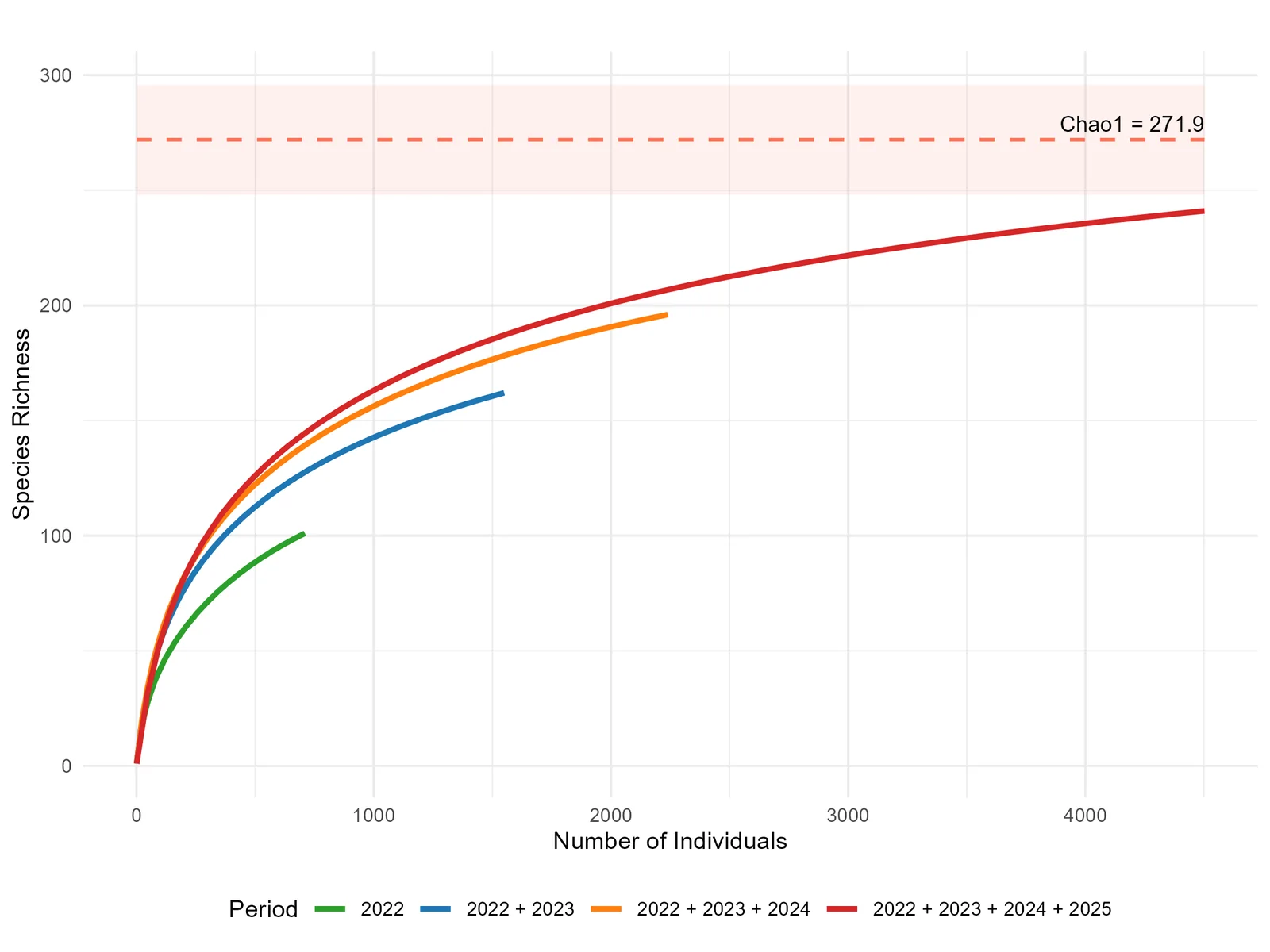
Species accumulation curves, based on cumulative sampling effort across four consecutive years (2022–2025), each restricted around ten-day period in mid-July. For each period, the rarefaction curve (solid line) represents the expected species richness as a function of the number of individuals sampled (x-axis). The dashed horizontal line indicates the total species richness estimated for the full dataset (2022–2025) using the Chao1 estimator, with the shaded band representing the associated 95% confidence interval.

**Figure 4.**
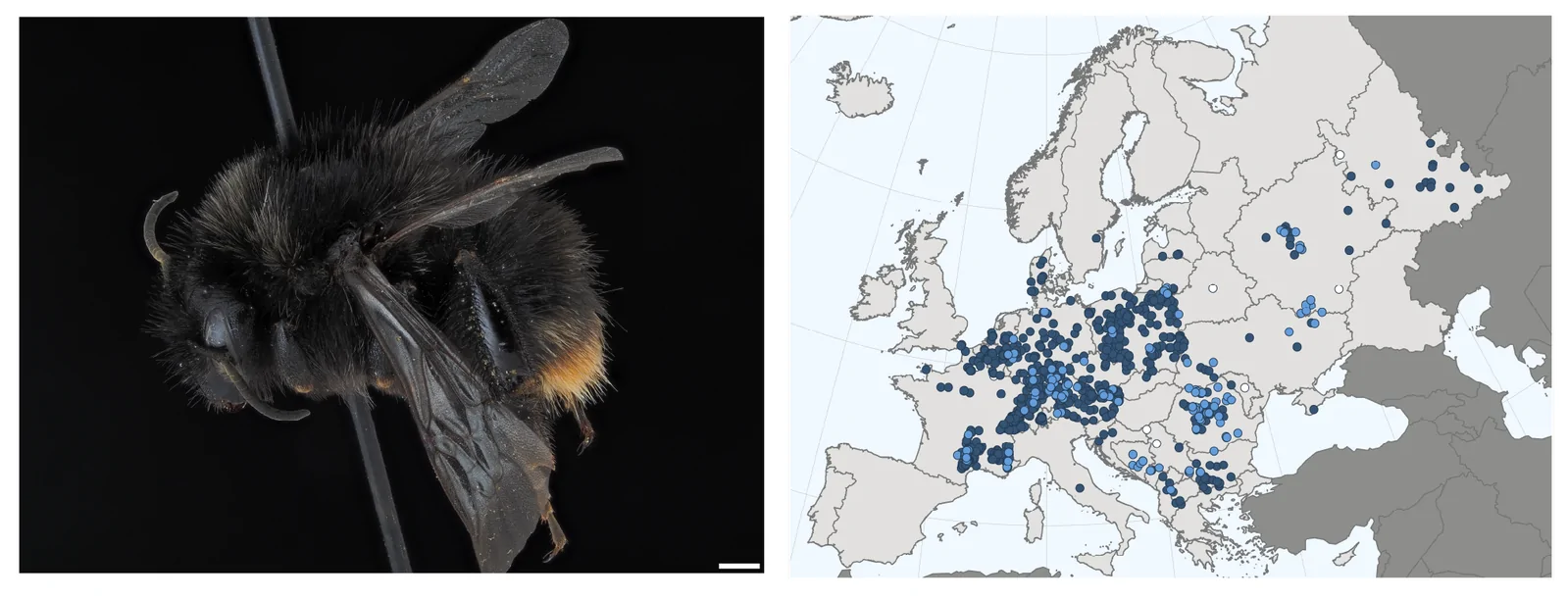
*Bombus pomorum* ♀. Habitus, lateral view (left); distribution map, based on Sentil et al. 2026 (right). Scale: 1 mm. Photo credit Frédéric Carion.

**Figure 5.**
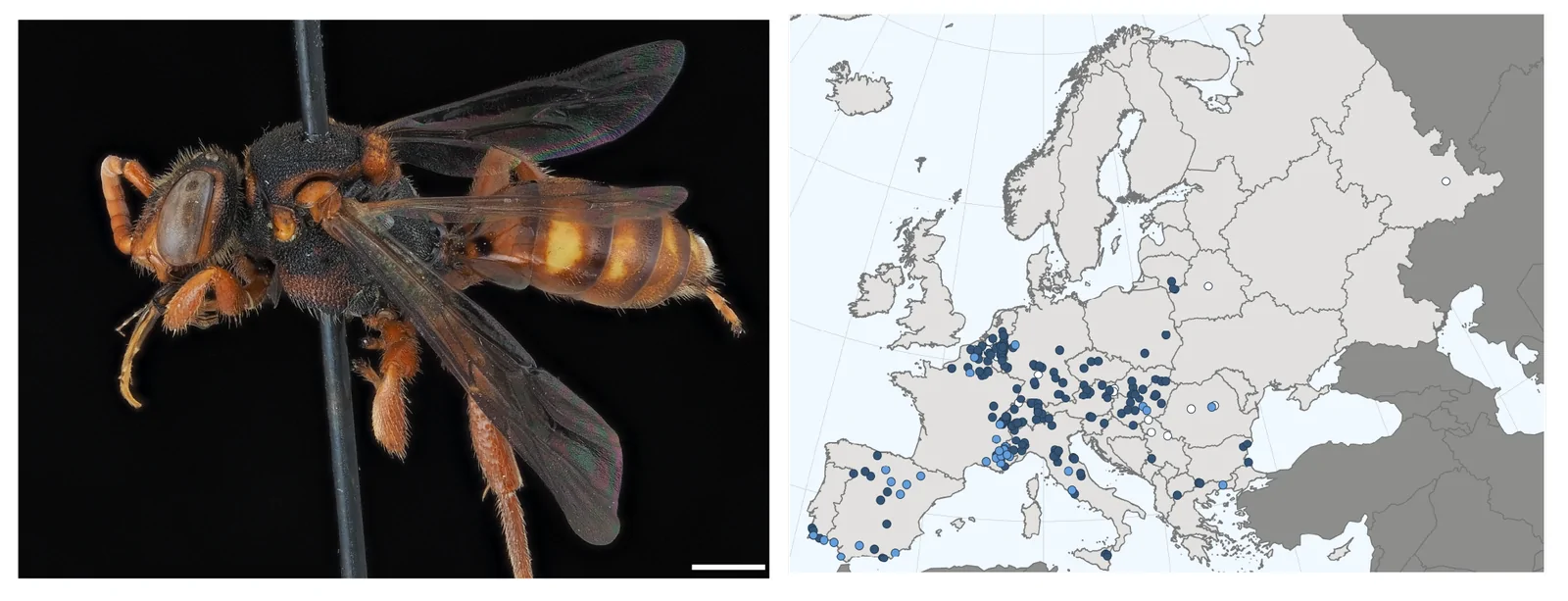
*Nomada rhenana* ♀. Habitus, lateral view (left); distribution map, based on Sentil et al. 2026 (right). Scale: 1 mm. Photo credit Frédéric Carion.

**Figure 6.**
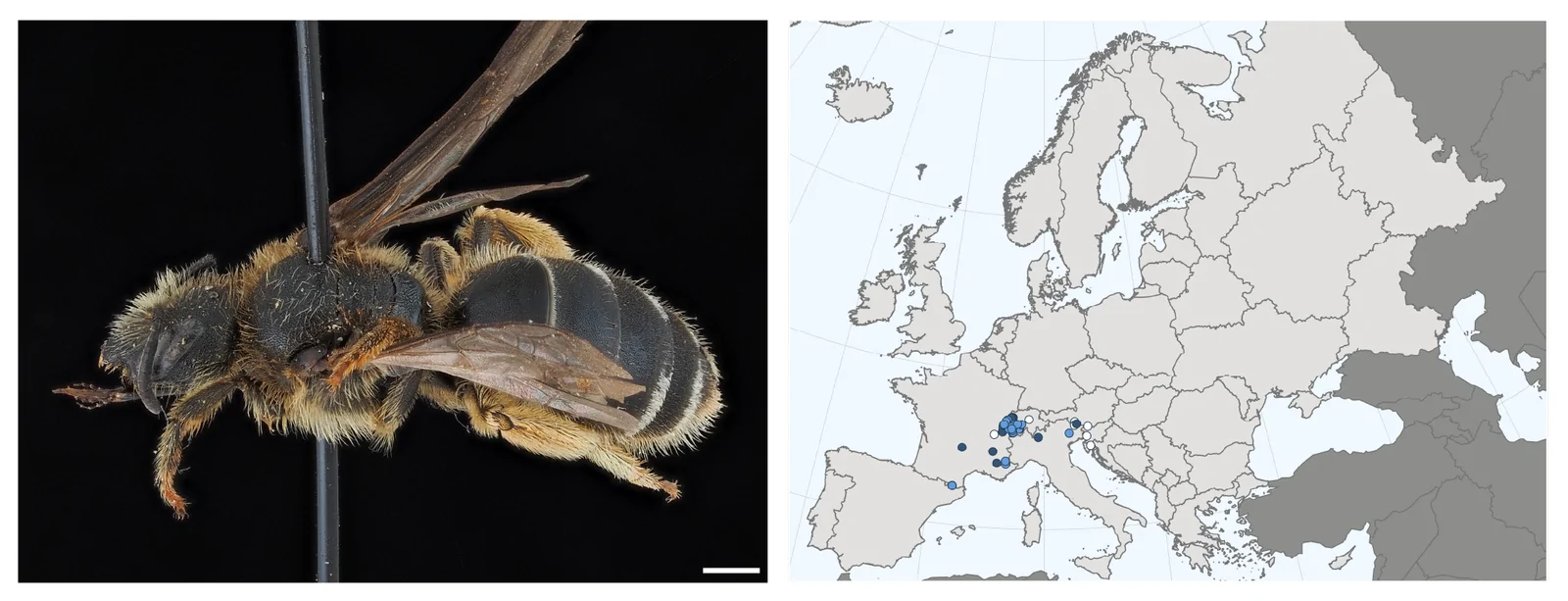
*Halictus carinthiacus* ♀. Habitus, lateral view (left); distribution map, based on Sentil et al. 2026 (right). Scale: 1 mm. Photo credit Frédéric Carion.

**Figure 7.**
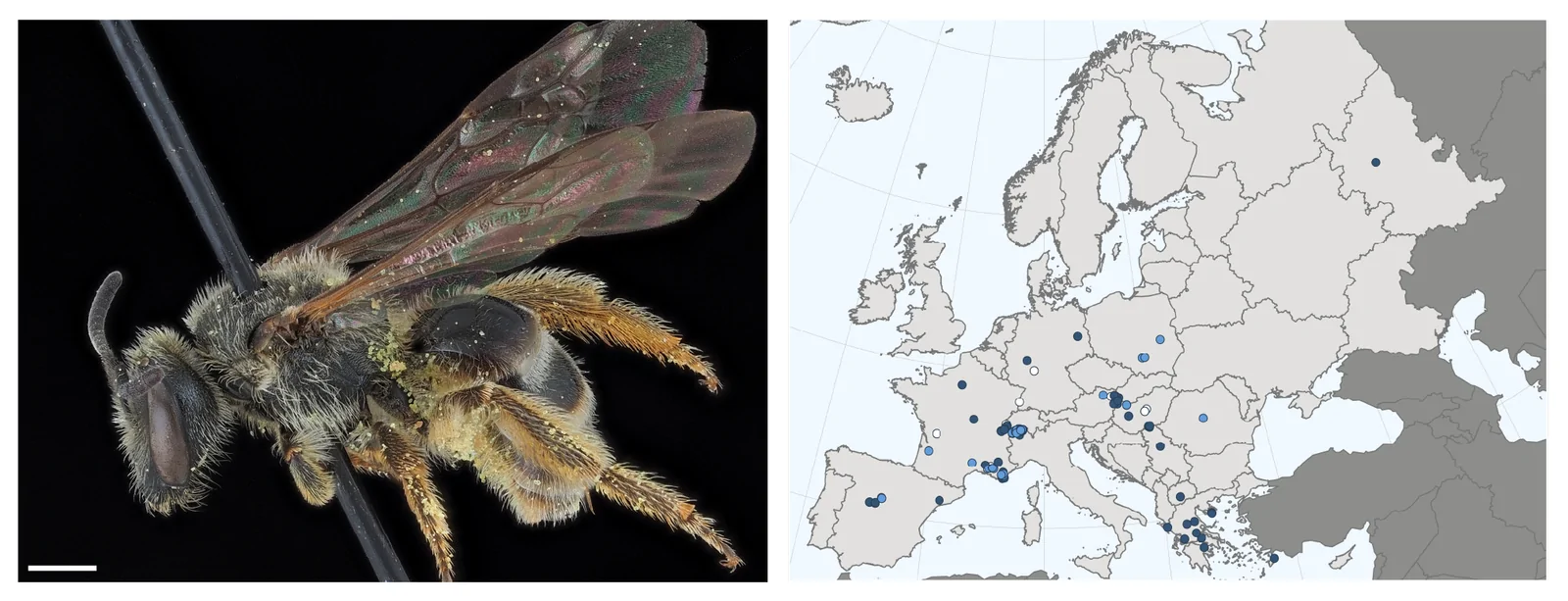
*Lasioglossum euboeense* ♀. Habitus, lateral view (left); distribution map, based on Sentil et al. 2026 (right). Scale: 1 mm. Photo credit Frédéric Carion.

**Figure 8.**
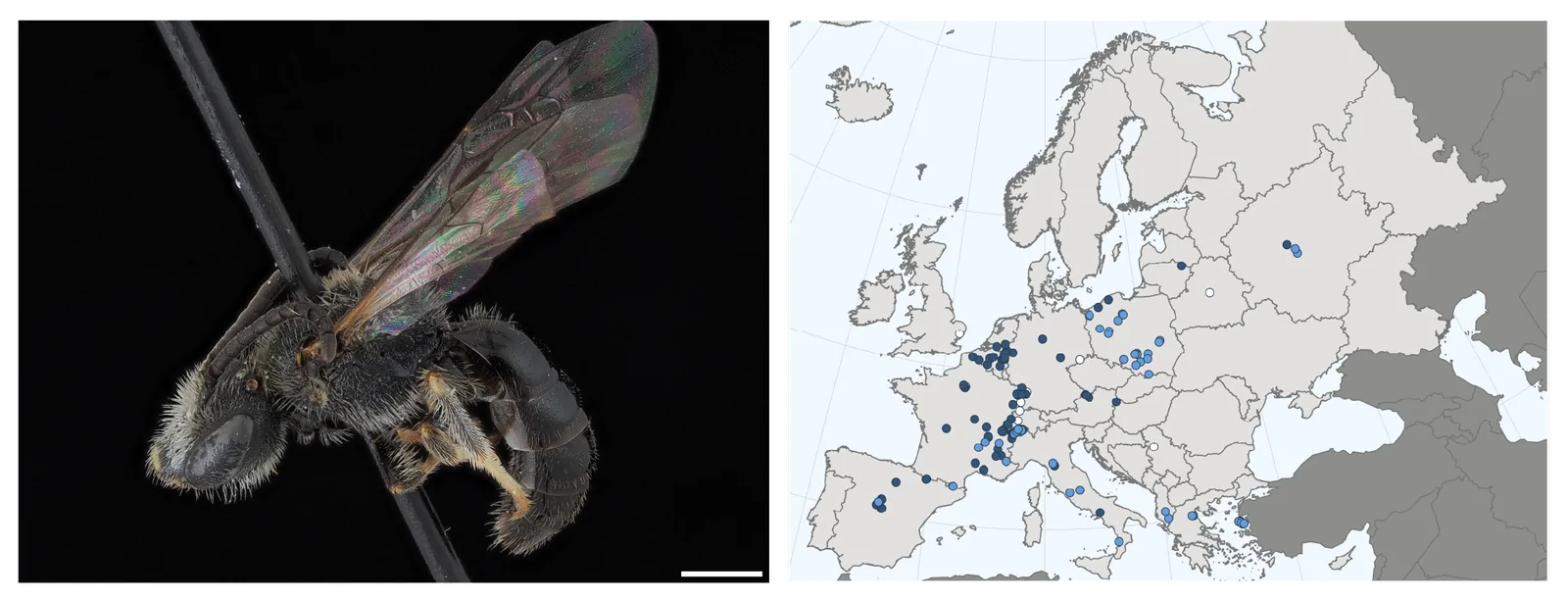
*Lasioglossum laeve* ♂. Habitus, lateral view (left); distribution map, based on Sentil et al. 2026 (right). Scale: 1 mm. Photo credit Frédéric Carion.

**Figure 9.**
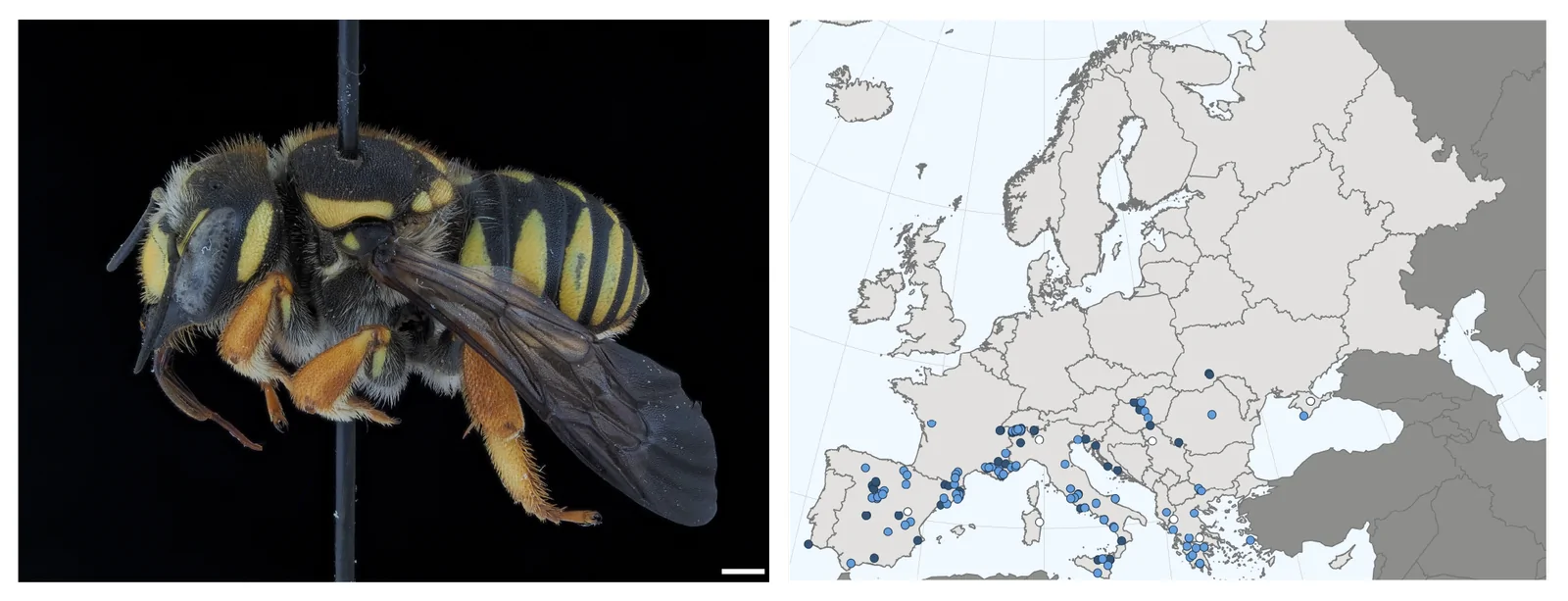
*Trachusa interrupta* ♀. Habitus, lateral view (left); distribution map, based on Sentil et al. 2026 (right). Scale: 1 mm. Photo credit Frédéric Carion.

**Figure 10.**
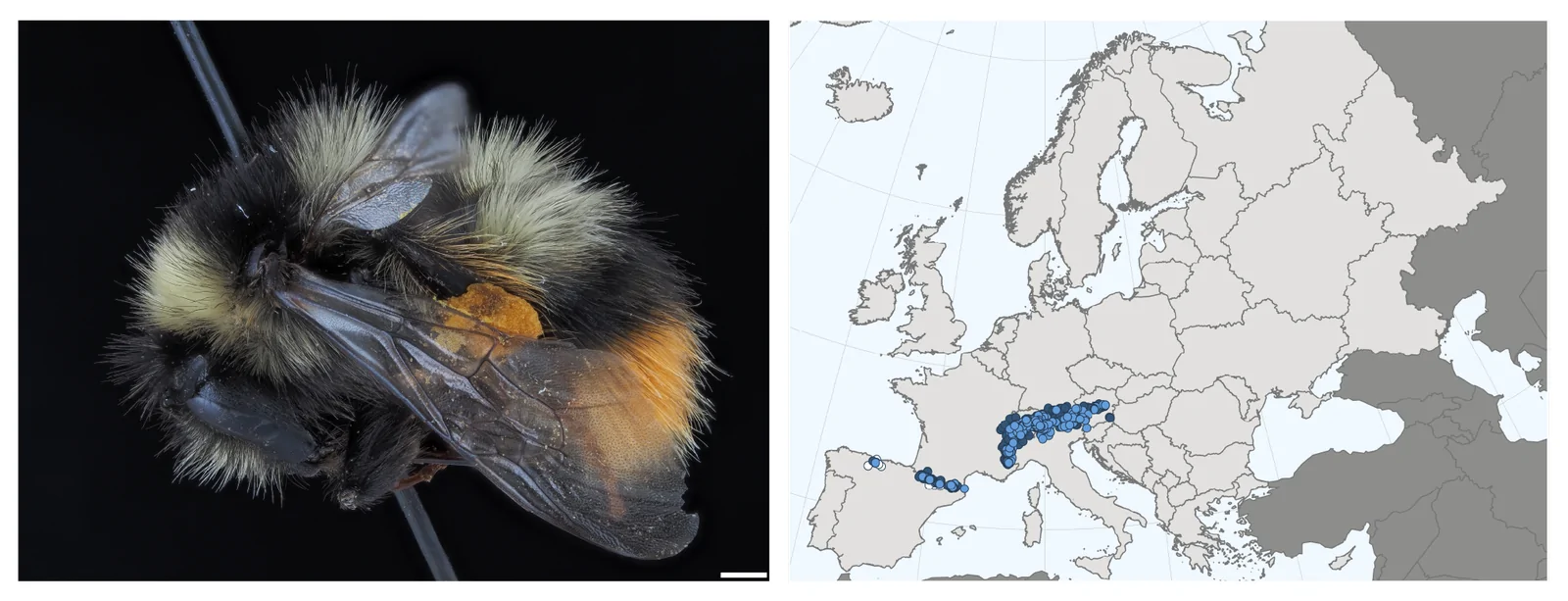
*Bombus mendax* ♀. Habitus, lateral view (left); distribution map, based on Sentil et al. 2026 (right). Scale: 1 mm. Photo credit Frédéric Carion.

**Figure 11.**
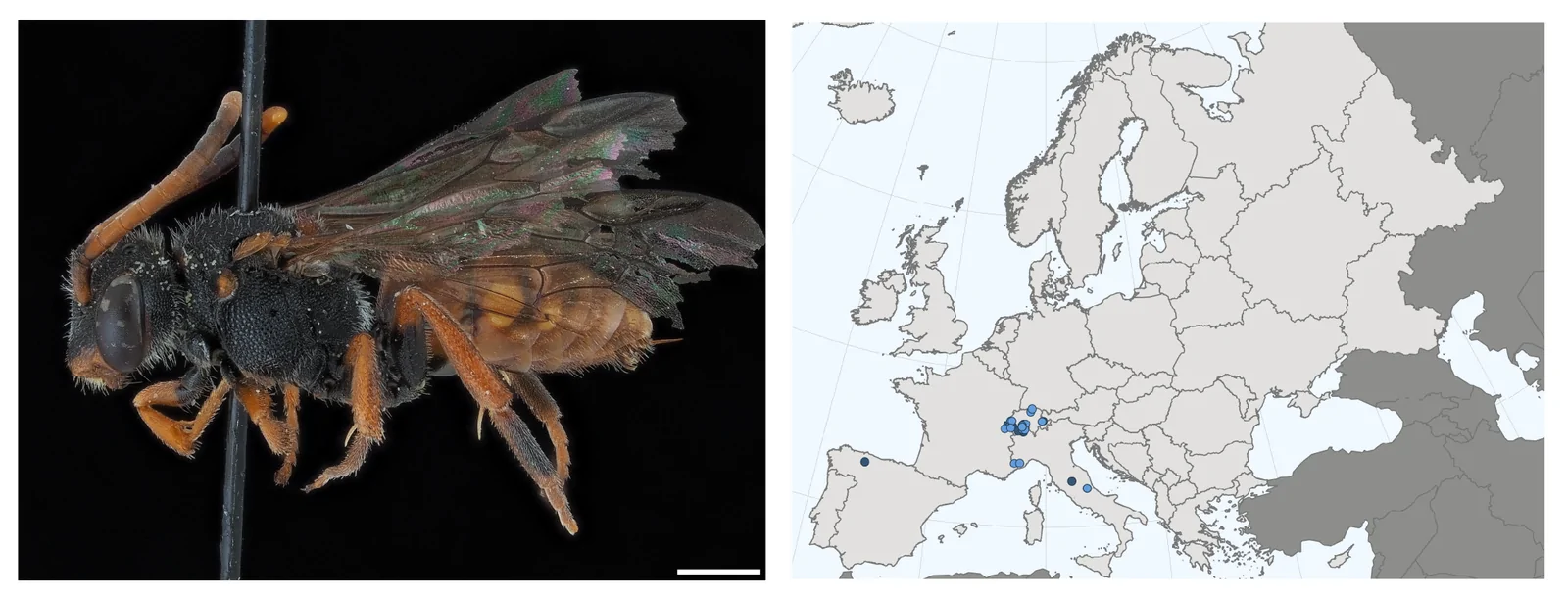
*Nomada gransassoi* ♀. Habitus, lateral view (left); distribution map, based on Sentil et al. 2026 (right). Scale: 1 mm. Photo credit Frédéric Carion.

**Figure 12.**
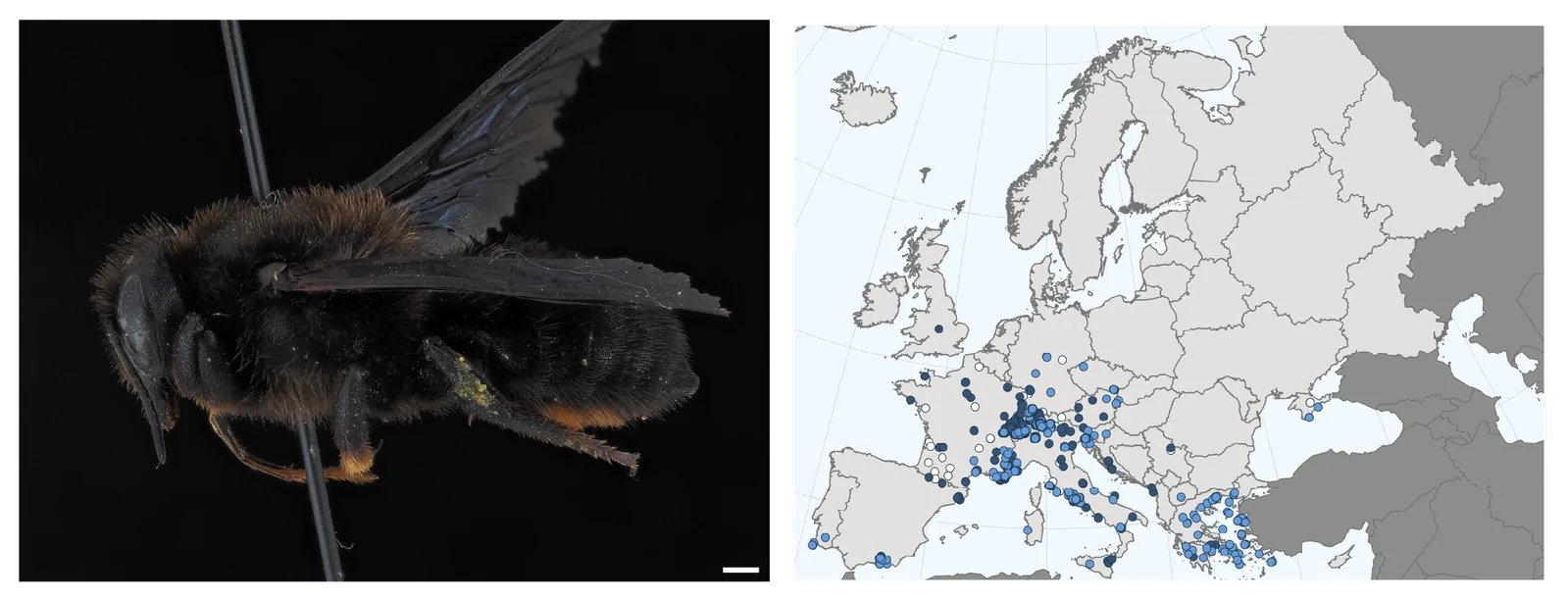
*Megachile parietina* ♀. Habitus, lateral view (left); distribution map, based on Sentil et al. 2026 (right). Scale: 1 mm. Photo credit Frédéric Carion.

**Figure 13.**
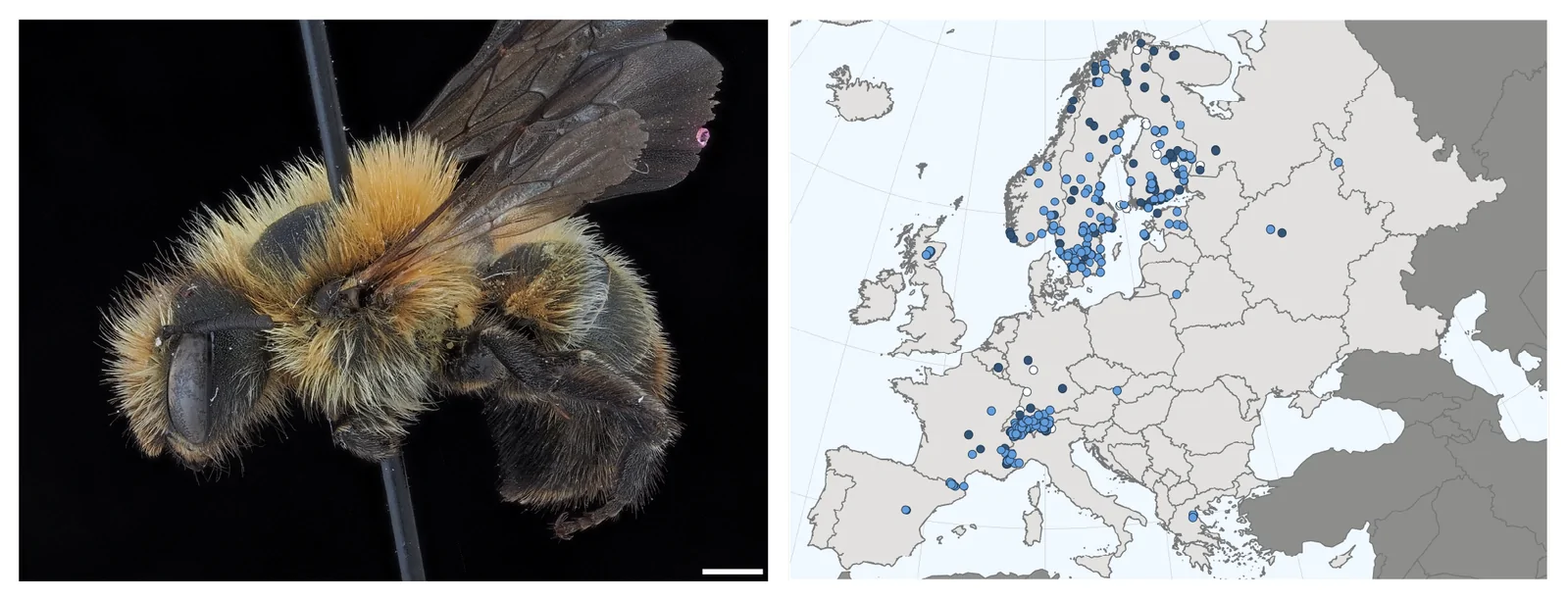
*Osmia inermis* ♀. Habitus, lateral view (left); distribution map, based on Sentil et al. 2026 (right). Scale: 1 mm. Photo credit Frédéric Carion.

**Table 1.** Summary of wild bee specimens collected in the Peyresq area (southern French Alps), grouped by family, with corresponding European IUCN Red List status across geographic Europe (Michez et al. 2026).

**Family**	**Scientific name**	**N**	**IUCN Europe**
Andrenidae	*Andrena afzeliella* (Kirby, 1802)	15	LC
	*Andrena amieti* Praz, Müller & Genoud, 2019	1	LC
	*Andrena ampla* Warncke, 1967	10	LC
	*Andrena barbareae* Panzer, 1805	3	LC
	*Andrena bicolor* Fabricius, 1775	30	LC
	*Andrena combinata* (Christ, 1791)	6	LC
	*Andrena congruens* Schmiedeknecht, 1884	4	LC
	*Andrena curvungula* Thomson, 1870	9	LC
	*Andrena falsifica* Perkins, 1915	5	LC
	*Andrena flavipes* Panzer, 1799	9	LC
	*Andrena fucata* Smith, 1847	2	LC
	*Andrena fulvago* (Christ, 1791)	14	LC
	*Andrena fulvicornis* (Schenck, 1853)	1	LC
	*Andrena haemorrhoa* (Fabricius, 1781)	1	LC
	*Andrena hattorfiana* (Fabricius, 1775)	2	LC
	*Andrena humilis* Imhoff, 1832	5	LC
	*Andrena intermedia* Thomson, 1870	67	LC
	*Andrena labialis* (Kirby, 1802)	33	LC
	*Andrena labiata* Fabricius, 1781	68	LC
	*Andrena limata* Smith, 1853	3	LC
	*Andrena minutula* (Kirby, 1802)	15	LC
	*Andrena minutuloides* Perkins, 1914	32	LC
	*Andrena montana* Warncke, 1973	1	LC
	*Andrena nigroaenea* (Kirby, 1802)	11	LC
	*Andrena ovatula* (Kirby, 1802)	6	LC
	*Andrena polita* Smith, 1847	11	LC
	*Andrena rufizona* Imhoff, 1834	21	NT
	*Andrena rufula* Schmiedeknecht, 1883	5	LC
	*Andrena russula* Lepeletier, 1841	11	LC
	*Andrena saxonica* Stöckhert, 1935	3	LC
	*Andrena schencki* Morawitz, 1866	1	LC
	*Andrena semilaevis* Pérez, 1903	2	LC
	*Andrena subopaca* Nylander, 1848	2	LC
	*Andrena tarsata* Nylander, 1848	4	LC
	*Andrena thomsonii* Ducke, 1898	1	DD
	*Andrena wilkella* (Kirby, 1802)	13	LC
	*Panurginus montanus* Giraud, 1861	4	LC
	*Panurgus banksianus* (Kirby, 1802)	14	LC
	*Panurgus dentipes* Latreille, 1811	120	LC
Apidae	*Amegilla garrula* (Rossi, 1790)	1	LC
	*Anthophora aestivalis* (Panzer, 1801)	67	LC
	*Anthophora balneorum* Lepeletier, 1841	1	LC
	*Anthophora furcata* (Panzer, 1798)	1	LC
	*Anthophora gallica* Dalla Torre & Friese, 1895	1	LC
	*Anthophora mucida* Gribodo, 1873	4	LC
	*Anthophora pubescens* (Fabricius, 1781)	1	LC
	*Bombus argillaceus* Scopoli, 1763	13	LC
	*Bombus barbutellus* (Kirby, 1802)	29	LC
	*Bombus bohemicus* Seidl, 1837	6	LC
	*Bombus brodmannicus* Vogt, 1909	4	NT
	*Bombus hortorum* (Linnaeus, 1761)	75	LC
	*Bombus humilis* Illiger, 1806	145	LC
	*Bombus hypnorum* (Linnaeus, 1758)	4	LC
	*Bombus lapidarius* (Linnaeus, 1758)	286	LC
	*Bombus lucorum* (Linnaeus, 1761)	43	LC
	*Bombus mastrucatus* Gerstäcker, 1869	35	LC
	*Bombus mendax* Gerstäcker, 1869	10	LC
	*Bombus mesomelas* Gerstäcker, 1869	54	LC
	*Bombus monticola* Smith, 1849	2	LC
	*Bombus mucidus* Gerstäcker, 1869	30	LC
	*Bombus pascuorum* (Scopoli, 1763)	120	LC
	*Bombus pomorum* (Panzer, 1805)	3	VU
	*Bombus pratorum* (Linnaeus, 1761)	129	LC
	*Bombus ruderarius* (Müller, 1776)	379	LC
	*Bombus rupestris* (Fabricius, 1793)	28	LC
	*Bombus* s. str. spp.	417	/
	*Bombus sichelii* Radoszkowski, 1859	17	LC
	*Bombus soroeensis* (Fabricius, 1777)	83	LC
	*Bombus subterraneus* (Linnaeus, 1758)	17	NT
	*Bombus sylvarum* (Linnaeus, 1761)	6	LC
	*Bombus sylvestris* (Lepeletier, 1832)	16	LC
	*Bombus vestalis* (Geoffroy, 1785)	2	LC
	*Ceratina chalybea* Chevrier, 1872	5	LC
	*Ceratina cucurbitina* (Rossi, 1792)	40	LC
	*Ceratina cyanea* (Kirby, 1802)	21	LC
	*Ceratina gravidula* Gerstaecker, 1869	2	LC
	*Epeolus julliani* Pérez, 1884	1	LC
	*Eucera longicornis* (Linnaeus, 1758)	25	LC
	*Eucera nigrescens* Pérez, 1879	1	LC
	*Melecta luctuosa* (Scopoli, 1770)	8	LC
	*Nomada facilis* Schwarz, 1967	3	LC
	*Nomada flavoguttata* (Kirby, 1802)	1	LC
	*Nomada gransassoi* Schwarz, 1986	1	LC
	*Nomada panurgina* Morawitz, 1869	1	LC
	*Nomada rhenana* Morawitz, 1872	1	VU
	*Tetralonia fulvescens* Giraud, 1863	2	LC
	*Tetralonia malvae* (Rossi, 1790)	1	LC
	*Xylocopa iris* (Christ, 1791)	2	LC
	*Xylocopa valga* Gerstaecker 1872	6	LC
	*Xylocopa violacea* (Linnaeus, 1758)	44	LC
Colletidae	*Colletes hylaeiformis* Eversmann, 1852	1	LC
	*Colletes nigricans* Gistel, 1857	2	LC
	*Hylaeus angustatus* (Schenck, 1861)	4	LC
	*Hylaeus clypearis* (Schenck, 1853)	3	LC
	*Hylaeus communis* Nylander, 1852	36	LC
	*Hylaeus confusus* Nylander, 1852	8	LC
	*Hylaeus difformis* (Eversmann, 1852)	4	LC
	*Hylaeus dilatatus* (Kirby, 1802)	1	LC
	*Hylaeus duckei* (Alfken, 1904)	1	LC
	*Hylaeus gibbus* Saunders, 1850	8	LC
	*Hylaeus hyalinatus* Smith, 1842	1	LC
	*Hylaeus imparilis* Förster, 1871	1	LC
	*Hylaeus incongruus* Förster, 1871	16	LC
	*Hylaeus kahri* Förster, 1871	35	LC
	*Hylaeus nigritus* (Fabricius, 1798)	24	LC
	*Hylaeus punctulatissimus* Smith, 1842	5	LC
	*Hylaeus purpurissatus* (Vachal, 1895)	2	LC
Halictidae	*Dufourea dentiventris* (Nylander, 1848)	2	NT
	Halictus aff. adjikenticus Blüthgen, 1923	79	/
	*Halictus alfkenellus* Strand, 1909	5	DD
	*Halictus brunnescens* (Eversmann, 1852)	4	LC
	*Halictus carinthiacus* Blüthgen, 1936	4	EN
	*Halictus eurygnathus* Blüthgen, 1930	2	LC
	*Halictus langobardicus* Blüthgen, 1944	13	LC
	*Halictus maculatus* Smith, 1848	35	LC
	*Halictus quadricinctus* (Fabricius, 1777)	24	LC
	*Halictus rubicundus* (Christ, 1791)	1	LC
	*Halictus scabiosae* (Rossi, 1790)	42	LC
	*Halictus sexcinctus* (Fabricius, 1775)	32	LC
	*Halictus simplex* Blüthgen, 1923	13	LC
	*Lasioglossum aeratum* (Kirby, 1802)	11	LC
	*Lasioglossum albipes* (Fabricius, 1781)	56	LC
	*Lasioglossum albocinctum* (Lucas, 1849)	1	LC
	*Lasioglossum angusticeps* (Perkins, 1895)	1	NT
	*Lasioglossum calceatum* (Scopoli, 1763)	29	LC
	*Lasioglossum convexiusculum* (Schenck, 1853)	9	NT
	*Lasioglossum costulatum* (Kriechbaumer, 1873)	22	LC
	*Lasioglossum crassepunctatum* (Blüthgen, 1923)	22	LC
	*Lasioglossum cupromicans* (Pérez, 1903)	2	LC
	*Lasioglossum euboeense* (Strand, 1909)	3	VU
	*Lasioglossum fratellum* (Pérez, 1903)	1	LC
	*Lasioglossum fulvicorne* (Kirby, 1802)	20	LC
	*Lasioglossum interruptum* (Panzer, 1798)	5	LC
	*Lasioglossum laeve* (Kirby, 1802)	2	VU
	*Lasioglossum laevigatum* (Kirby, 1802)	6	LC
	*Lasioglossum laticeps* (Schenck, 1870)	38	LC
	*Lasioglossum lativentre* (Schenck, 1853)	5	LC
	*Lasioglossum leucopus* (Kirby, 1802)	12	LC
	*Lasioglossum leucozonium* (Schrank, 1781)	5	LC
	*Lasioglossum marginellum* (Schenck, 1853)	2	NT
	*Lasioglossum medinai* (Vachal, 1895)	2	LC
	*Lasioglossum morio* (Fabricius, 1793)	31	LC
	*Lasioglossum nigripes* (Lepeletier, 1841)	10	LC
	*Lasioglossum nitidulum* (Fabricius, 1804)	7	LC
	*Lasioglossum parvulum* (Schenck, 1853)	6	LC
	*Lasioglossum pauperatum* (Brullé, 1832)	1	LC
	*Lasioglossum pauxillum* (Schenck, 1853)	17	LC
	*Lasioglossum politum* (Schenck, 1853)	5	LC
	*Lasioglossum punctatissimum* (Schenck, 1853)	1	LC
	*Lasioglossum pygmaeum* (Schenck, 1853)	1	LC
	*Lasioglossum quadrinotatum* (Kirby, 1802)	2	LC
	*Lasioglossum sexnotatum* (Kirby, 1802)	1	LC
	*Lasioglossum subhirtum* (Lepeletier, 1841)	44	LC
	*Lasioglossum tricinctum* (Schenck, 1874)	59	LC
	*Lasioglossum villosulum* (Kirby, 1802)	3	LC
	*Lasioglossum xanthopus* (Kirby, 1802)	18	LC
	*Seladonia gavarnica* (Pérez, 1903)	2	LC
	*Seladonia subaurata* (Rossi, 1792)	1	LC
	*Seladonia tumulorum* (Linnaeus, 1758)	12	LC
	*Sphecodes albilabris* (Fabricius, 1793)	1	LC
	*Sphecodes crassus* Thomson, 1870	1	LC
	*Sphecodes ephippius* (Linnaeus, 1767)	8	LC
	*Sphecodes ferruginatus* von Hagens, 1882	8	LC
	*Sphecodes gibbus* (Linnaeus, 1758)	3	LC
	*Sphecodes majalis* Pérez, 1903	9	LC
	*Sphecodes monilicornis* (Kirby, 1802)	18	LC
	*Sphecodes niger* von Hagens, 1874	3	LC
	*Sphecodes puncticeps* Thomson, 1870	4	LC
	*Sphecodes reticulatus* Thomson, 1870	1	LC
Megachilidae	*Anthidiellum breviusculum* (Pérez, 1890)	6	LC
	*Anthidiellum strigatum* (Panzer, 1805)	4	LC
	*Anthidium manicatum* (Linnaeus, 1758)	4	LC
	*Anthidium oblongatum* (Illiger, 1806)	4	LC
	*Anthidium punctatum* Latreille, 1809	28	LC
	*Chelostoma campanularum* (Kirby, 1802)	20	LC
	*Chelostoma distinctum* (Stoeckhert, 1929)	6	LC
	*Chelostoma florisomne* (Linnaeus, 1758)	41	LC
	*Chelostoma foveolatum* (Morawitz, 1868)	45	LC
	*Chelostoma grande* (Nylander, 1852)	45	NT
	*Chelostoma rapunculi* (Lepeletier, 1841)	42	LC
	*Coelioxys conicus* (Linnaeus, 1758)	1	LC
	*Heriades truncorum* (Linnaeus, 1758)	59	LC
	*Hoplitis acuticornis* (Dufour & Perris, 1840)	19	LC
	*Hoplitis adunca* (Panzer, 1798)	35	LC
	*Hoplitis anthocopoides* (Schenck, 1853)	8	LC
	*Hoplitis brachypogon* (Pérez, 1879)	3	LC
	*Hoplitis claviventris* (Thomson, 1872)	6	LC
	*Hoplitis lepeletieri* (Pérez, 1879)	6	LC
	*Hoplitis leucomelana* (Kirby, 1802)	8	LC
	*Hoplitis loti* (Morawitz, 1867)	2	LC
	*Hoplitis mitis* (Nylander, 1852)	2	LC
	*Hoplitis papaveris* (Latreille, 1799)	5	LC
	*Hoplitis praestans* (Morawitz, 1893)	6	LC
	*Hoplitis ravouxi* (Pérez, 1902)	1	LC
	*Hoplitis villosa* (Schenck, 1853)	1	LC
	*Lithurgus chrysurus* Fonscolombe, 1834	8	LC
	*Megachile alpicola* Alfken, 1924	2	LC
	*Megachile analis* Nylander, 1852	2	LC
	*Megachile argentata* (Fabricius, 1793)	5	LC
	*Megachile centuncularis* (Linnaeus, 1758)	2	LC
	*Megachile circumcincta* (Kirby, 1802)	32	LC
	*Megachile ericetorum* (Lepeletier, 1841)	6	LC
	*Megachile genalis* Morawitz, 1880	1	LC
	*Megachile lagopoda* (Linnaeus, 1761)	5	LC
	*Megachile maritima* (Kirby, 1802)	2	LC
	*Megachile octosignata* Nylander, 1852	1	LC
	*Megachile parietina* (Geoffroy, 1785)	37	LC
	*Megachile pyrenaica* (Lepeletier, 1841)	13	LC
	*Megachile rufescens* (Pérez, 1879)	3	LC
	*Megachile sculpturalis* Smith, 1853	4	NA
	*Megachile versicolor* Smith, 1844	1	LC
	*Megachile willughbiella* (Kirby, 1802)	38	LC
	*Osmia andrenoides* Spinola, 1808	1	LC
	*Osmia argyropyga* Pérez, 1879	4	LC
	*Osmia aurulenta* (Panzer, 1799)	58	LC
	*Osmia bicornis* (Linnaeus, 1758)	5	LC
	*Osmia caerulescens* (Linnaeus, 1758)	3	LC
	*Osmia cerinthidis* Morawitz, 1875	3	LC
	*Osmia cyanoxantha* Pérez, 1879	12	LC
	*Osmia emarginata* Lepeletier, 1841	1	LC
	*Osmia gallarum* Spinola, 1808	6	LC
	*Osmia inermis* (Zetterstedt, 1838)	5	LC
	*Osmia labialis* Pérez, 1879	2	LC
	*Osmia leaiana* (Kirby, 1802)	23	LC
	*Osmia niveata* (Fabricius, 1804)	14	LC
	*Osmia niveocincta* Pérez, 1897	7	LC
	*Osmia parietina* Curtis, 1828	2	LC
	*Osmia rufohirta* Latreille, 1811	2	LC
	*Osmia spinulosa* (Kirby, 1802)	4	LC
	*Osmia submicans* Morawitz, 1870	19	LC
	*Osmia tergestensis* Ducke, 1897	11	LC
	*Osmia xanthomelana* (Kirby, 1802)	7	LC
	*Pseudoanthidium nanum* (Mocsáry, 1880)	6	LC
	*Rhodanthidium caturigense* (Giraud, 1863)	3	LC
	*Stelis breviuscula* (Nylander, 1848)	1	LC
	*Stelis nasuta* (Latreille, 1809)	1	NT
	*Stelis punctulatissima* (Kirby, 1802)	2	LC
	*Stelis signata* (Latreille, 1809)	2	LC
	*Trachusa byssina* (Panzer, 1798)	19	LC
	*Trachusa interrupta* (Fabricius, 1781)	2	VU
Melittidae	*Melitta haemorrhoidalis* (Fabricius, 1775)	10	LC
Total		4502	
